# Enhancing severely compromised premolar strength: role of cusp reduction design in CAD/CAM composite restorations

**DOI:** 10.1007/s10266-025-01167-5

**Published:** 2025-08-16

**Authors:** Mohamed F. Haridy, Ahmed Refaat Mohamed, Shehabeldin Saber, Edgar Schafer, Samar Elsayed Swelam, Youssef M. Haridy, Hend S. Ahmed

**Affiliations:** 1 Department of Conservative Dentistry, Faculty of Dentistry, The British University in Egypt, Al Shorouk city, Cairo, Egypt; 2 Department of Conservative Dentistry, Faculty of Dentistry, Cairo University, Cairo, Egypt; 3 Department of Endodontics, Faculty of Dentistry, The British University in Egypt, Al Shorouk city, Cairo, Egypt; 4 Department of Endodontics, Faculty of Dentistry, Ain Shams University, Cairo, Egypt; 5 Central Interdisciplinary Ambulance in the School of Dentistry, University of Münster, Münster, Germany; 6 Third Settlement Medical Center, Ministry of Health and Population, Cairo, Egypt; 7 Dental Student at Faculty of Dentistry, The British University in Egypt, Al Shorouk city, Cairo, Egypt

**Keywords:** Cad/Cam, Composite block, Inlay, Overlay, Indirect restoration

## Abstract

The objective of this study was to assess the effect of different designs and depths of cusp reduction on fracture resistance of maxillary permanent premolars restored with CAD/CAM composite restorations. A total of 42 sound maxillary premolars were used. Standardized MOD cavities were prepared in all specimens. Teeth were randomly divided according to form of cusp reduction into three main groups (*n* = 14); G1:MOD cavities restored with inlays with no cusp reduction, G2:MOD cavities restored with overlays with buccal and palatal anatomical cusps reduction, G3; MOD cavities restored with overlays with buccal and palatal flat cusps reduction. Groups 2 and 3 were further sub-divided into two sub-groups (*n* = 7) according to amount of cusp reduction either 1.5 mm or 2.5 mm. All groups were restored by CAD/CAM resin composite indirect restorations and cemented by adhesive resin cement. Thermocycling was done for all specimens. Fracture resistance was tested by universal testing machine and failure modes were examined by stereomicroscope. Statistically analysis was done for all data. Fracture resistance showed significant differences among the experimental groups (*p* < 0.001) with the highest fracture resistance for overlays with 2.5 mm of anatomical reduction. Regarding the modes of failure, there were no significant differences between experimental groups (*p* = 0.489). The fracture resistance of the composite CAD/CAM fabricated restorations is highly influenced by the restoration/prep design. The overlay design with anatomical cusps reduction of 2.5 mm can reinforce maxillary premolars teeth with MOD cavities.

## Introduction

The aim of restorative dentistry is to preserve natural teeth while ensuring optimal function and attractive aesthetics. Caries, trauma, and cavity preparations lead to loss of tooth structure which decrease the fracture resistance of the restored tooth. Among all factors, “extensive cavity preparations” are the most common cause of tooth friability. Removal of marginal ridges, occlusal enamel, and cusps reduction are associated with a progressive decrease in fracture resistance [1, 2].

It has been stated that loss of one wall during cavity preparation along with an isthmus width of approximately one-third of the inter-cuspal distance decrease tooth fracture resistance by 20%. While cavity preparations destroying one or both marginal ridges resulted in loss of tooth stiffnes**s** by 46% and 63%, respectively [3]. Therefore, it is a big challenge to regain the fracture resistance of teeth after extensive cavity preparations.

Maxillary premolars exhibit a high incidence and the greatest vulnerability to fractures during functional loading [4]. This susceptibility is due to their narrow cervical thickness, a concavity on the mesial surface of the root, and a radicular groove on the palatal side of the buccal root, all of which contribute to a higher risk of cusp fractures, wedging, and splitting [5].

Consequently, when restoring mutilated vital maxillary premolars, full coverage restorations are not regarded as a conservative treatment option. Instead, overlay restorations with a minimum cusp reduction of 1.5 mm are recommended to safeguard both the tooth structure and the restoration from functional overload. In addition, minimizing lateral forces is strongly advised to lower the risk of fracture [6].

Previous studies assessed the impact of the amount and form of occlusal reduction on the clinical outcomes of overlay restorations [7, 8]. However, there is yet no consensus regarding the ideal occlusal reduction thickness and form on the tooth-restoration complex [9], especially with the modern restorative approaches.

Modern prosthetic dentistry is increasingly embracing digital workflows, particularly those involving CAD/CAM technologies. Ceramics and composites have shown great promise as materials for indirect restorations fabricated using these systems [10]. Among these, resin-based composite (RBC) blocks are gaining attention for their potential to revolutionize restorative treatments [11]. Indirect resin restorations offer several advantages, including excellent marginal integrity, precise proximal contacts, high wear resistance, reduced polymerization shrinkage, aesthetic appeal, and ease of fabrication and repair [7, 12]. Mechanically, these composite materials often contain a filler volume exceeding 70%, which enhances fracture resistance [13]. In addition, they demonstrate improved tensile and compressive strength, hardness, wear resistance, and elastic modulus. As a result, these materials are less prone to catastrophic failures, chipping, and crack formation during both manufacturing and clinical function [14].

Therefore, this study aimed to evaluate the fracture resistance of maxillary premolars restored with CAD/CAM composite restorations using various cusp reduction designs. The null hypothesis stated that there would be no statistically significant differences in fracture resistance between the different preparation designs (inlay and overlay), nor between the variations in the amount and configuration of cuspal reduction.

## Materials and methods

All materials’ specification, compositions, and manufacturers are summarized in Table 1.

**Table 1:** Materials’ specification, compositions, manufacturers, and LOT numbers

Material	Specifications	Composition	Manufacturer	Lot number
Brilliant Crios	Nano hybrid Resin Composite Block	Dental glass Barium glass Size < 1.0 µm, Amorphous silica SiO2 Size < 20 nm, Resin matrix Cross-linked methacrylates and Inorganic pigments, such as ferrous oxide or titanium dioxide	Coltene, Whaledent, Langenau, Germany	K56598
Aluminum Oxide	Abrasive powder	Aluminum Oxide 29 µm particle size	Velopex international, UK	100,119
FineEtch 37%	Acid etching gel	Phosphoric acid gel 37%	SPIDENT, Gojan-dong, Namdong-gu, Incheon, South Korea	FE21470
Porcelain primer	Silane coupling agent	Acetone, 3-(Trimethoxysilyl) Propyl 2-Propenoic Acid, Acetic Acid	Bisco, Schaumburg, IL, USA	2,200,003,351
Prime & Bond Universal Adhesive	Universal adhesive	Urethane dimethacrylate 2-hydroxyethyl methacrylate Photoinitiators Ethanol, Ethyl alcohol Water	Dentsply Sirona, Bensheim, Germany	21,060
Calibra Universal	Self-adhesive dual-cure resin cement	Dimethacrylate Resins; Camphorquinone (CQ) Photoinitiator; Stabilizers; Glass Fillers; Fumed silica; Titanium Dioxide; Pigments	Dentsply Sirona, Bensheim, Germany	00100279

### Sample size calculation

A power analysis was conducted to ensure sufficient statistical power for testing the null hypothesis that there is no difference in fracture resistance among various forms and amounts of cusp reduction in CAD/CAM composite blocks used in maxillary permanent premolars. An alpha level of 0.05 and a beta level of 0.2 (i.e., 80% power) were adopted, with an effect size of 1.28, calculated based on the findings of Alassar et al. [6]. The estimated required sample size was 33. To account for potential sample loss during testing, the sample size was increased by 30%, resulting in a final sample size of 42. The calculation was performed using G*Power software version 3.1.9 (Heinrich-Heine University, Düsseldorf, Germany).

### Teeth selection

The study was conducted after approval of the committee of ethics at the faculty of dentistry at the British University in Egypt (approval no. 21-023). Premolars were obtained from an Egyptian population with an age range of 16–25 years admitted to the oral and maxillofacial surgery department at the British university in Egypt. A total of 42 sound human first maxillary premolars freshly extracted for orthodontic treatment were selected. All patients approved their extracted teeth to be used in research and signed a consent explaining this. The teeth were evaluated to ensure anatomical uniformity in terms of crown length, as well as mesiodistal (7 ± 0.5 mm) and buccolingual (8 ± 0.5 mm) dimensions [15]. All dimensional were measured at the proximal cementoenamel junction using a digital caliper (Bacolis Digital Clipper, Stainless Hardened, Generic, China). Immediately following extraction, the teeth were cleaned, disinfected [16], and examined under magnification to ensure they were free of caries, cracks, or hypoplastic defects. Any teeth showing such defects were excluded and replaced. The selected specimens were stored in glass jars containing distilled water at 4 °C and used within 3 months [17]. The distilled water was refreshed every 5 days until the experiment commenced.

### Preparation of specimens and cavity preparation

Each tooth was fixed in cold-cured acryl (Acrostone, Cairo, Egypt) that was applied in plastic cylinders (3 cm × 2 cm). For periodontal ligament simulation, the roots of all specimens were covered by molten wax (Renfert GEO Classic, Hilzingen, Germany) to form a 0.2 mm to 0.3 mm film [18]. Then the teeth were implanted in an auto-polymerizing acrylic resin vertically till 2 mm below the C.E.J [13] while being in the soft dough stage. A centralizing device was used to ensure that the teeth were positioned with their long axis perpendicular to the base of the cylinder. The wax film was carefully removed by hot water and a wax blade. Light-body polyether impression material was then injected (Elite HD + , Zhermack, Rovigo Italy) into the obtained space and teeth were replaced in the block allowing the light body to set [19].

**Fig. 1:**
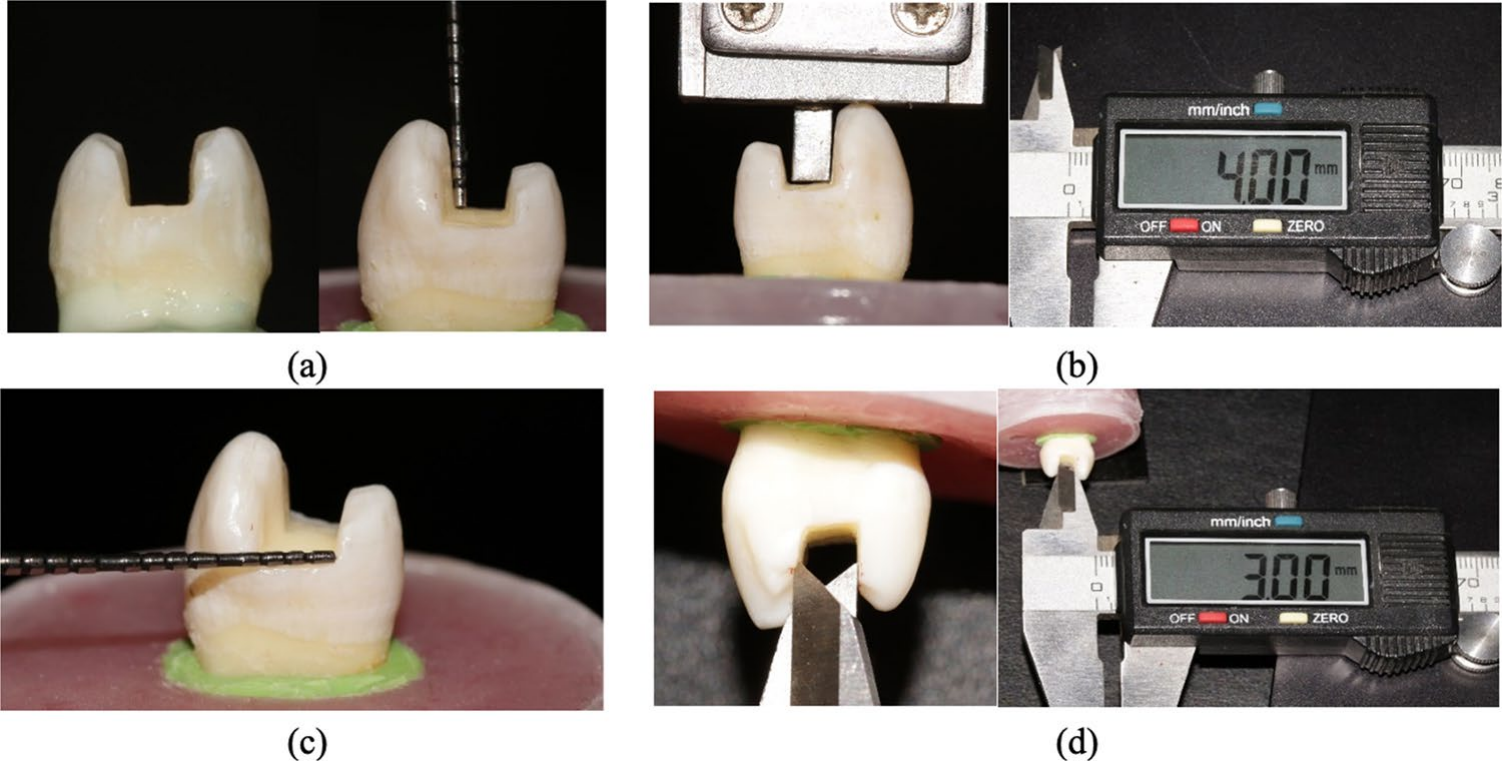
Groups I, II and III (**a**) MOD cavity depth 4 mm by periodontal probe, (**b**) MOD cavity depth 4 mm by digital clipper, (**c**) MOD cavity width 3 mm by periodontal probe, (**d**) MOD cavity width 3 mm by digital clipper

Standardized MOD preparations in all teeth without gingival seats. The cavity was prepared using a high-speed air–water cooling handpiece and #4261 inlay preparation kit (Komet Inlay preparation Kit, Brasseler, GmbH, Germany). Abrasives were replaced every four times of use. The prepared cavities had a depth of four millimeters measured from the buccal cusp tip as a reference point and a width of 3 mm representing one-half the inter-cuspal distance. This was measured using a periodontal probe and verified using a digital caliper (Fig. 1). To ensure standardization and repeatability of preparations, all preparations were done by the same operator.

### Grouping of specimens

**Fig. 2:**
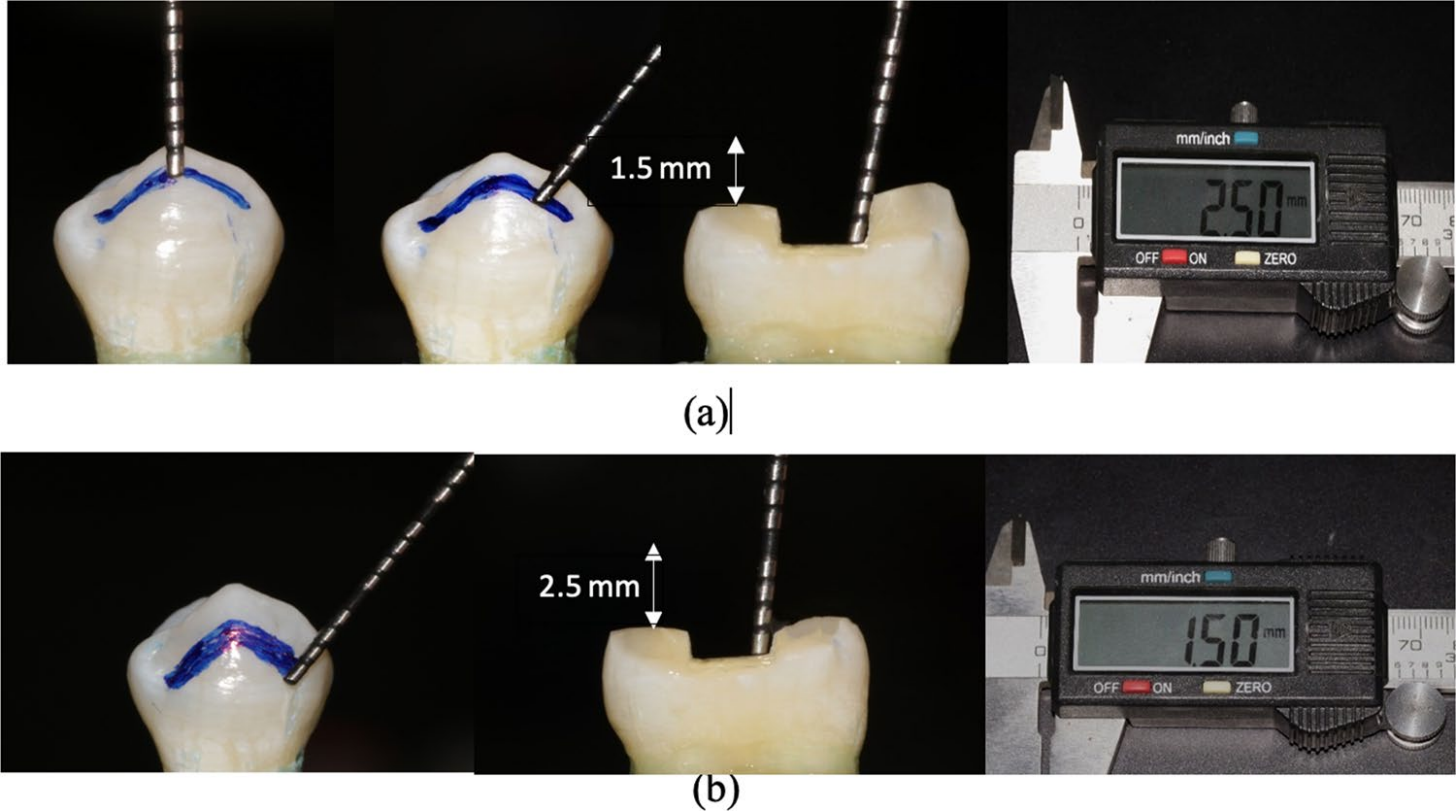
(**a**) *Group IIa* anatomical cusp reduction of 1.5 mm with remaining cavity depth 2.5 mm, (**b**) *Group IIb* anatomical cusp reduction of 2.5 with remaining cavity depth 1.5 mm

**Fig. 3:**
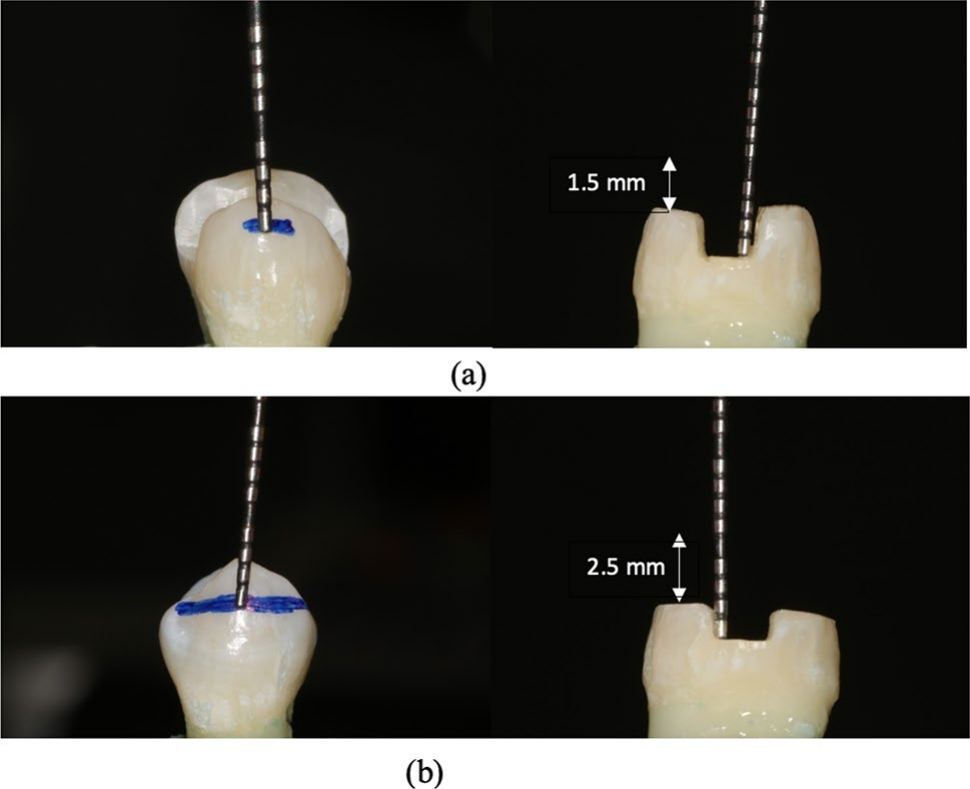
(**a**) *Group IIIa* flat cusp reduction of 1.5 mm with remaining cavity depth 2.5 mm, (**b**) Group *IIIb* flat cusp reduction of 2.5 mm with remaining cavity depth 1.5 mm

Random distribution of teeth was done according to the form of cusp reduction into three main groups (*n* = 14); G1:MOD cavities restored with inlays without cusp preparation, G2:MOD cavities restored with overlays with buccal and palatal anatomical cusps reduction (Fig. 2), G3; MOD cavities restored with overlays with buccal and palatal flat cusps reduction (Fig. 3). Groups 2 and 3 were further sub-divided into two sub-groups (*n* = 7) according to the amount of cusp reduction to be either 1.5 mm or 2.5 mm of cusps reduction.

### Construction of CAD/CAM composite restorations

All prepared specimens were scanned using the Omnicam intra-oral scanner integrated with the CEREC system (CEREC SW5, Dentsply Sirona) to capture digital impressions [15]. The scanning duration was ranged from 25 to 35 s for all specimens for standardization. The optical impressions were tested to avoid improper images that would affect the accuracy of the final restorations and then sent to the lab for designing. The design of the restorations was done using Exocad (Exocad, Darmstadt, Germany). The thickness of all restorations was standardized by the software. After designing the restoration, the margins, anatomy and contour were checked. The selected composite blocks were inserted in the milling machine (MC X5, Dentsply Sirona) and fixed with the set screw then milled and checked for accuracy and seating on their specimens**.** All specimens were polished in accordance with the manufacturer's guidelines using a Vita Enamic kit (VITA Zahnfabrik, Bad Säckingen, Germany), beginning with the coarsest grit tips and progressing to the finest.

### *Pretreatment of CAD/CAM composite restorations before cementation *(Fig. 4)

**Fig. 4:**
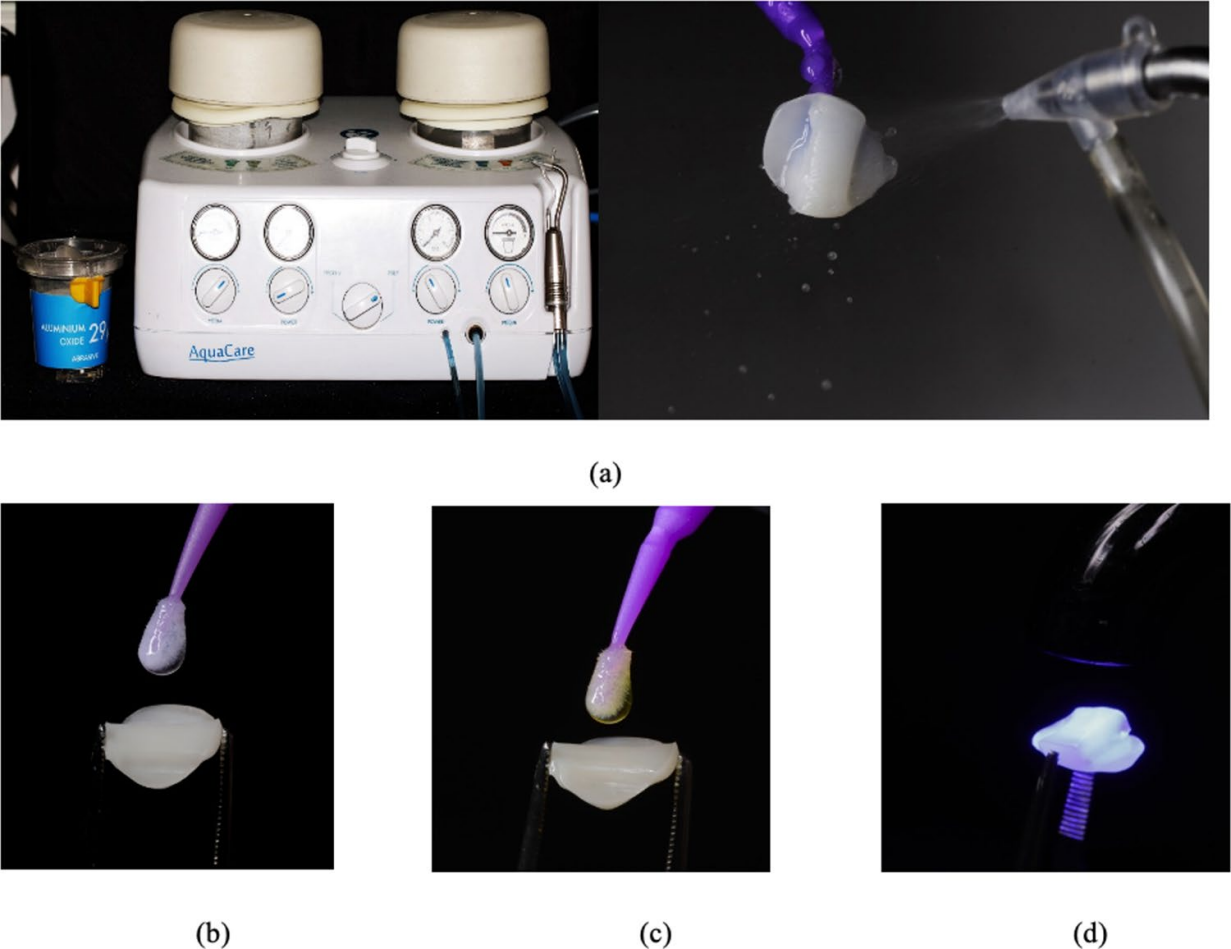
(a) Sandblasting using Aquacare dental air abrasion, (b) silane coupling agent, (c) universal adhesive prime and bond, (d) Light curing using light-emitting diode (LED)

The fitting surfaces of the final composite restorations underwent the following treatment: they were aluminablasted for 20 s using 29 µm aluminum oxide particles at an air pressure of 0.2 MPa [20], employing an Aquacare Twin Dental air abrasion unit (Velopex Int, Medivance Instruments, London, UK). After aluminablasting, the restorations were cleansed in an ultrasonication unit for 2 min, then rinsed for 20 s and dried by air for 10 s. A silane coupling agent was applied and left in place for 1 min before being air-dried [21]. Subsequently, a slim film of universal adhesive (Prime & Bond Universal, Dentsply Sirona, USA) was actively applied using a 1.5 mm green fine-type micro-brush (Easyinsmile, Passaic, New Jersey, USA) and left undisturbed for 20 s. The adhesive was then air-thinned for 10 s and cured for 20 s with an LED polymerization device at a light irradiance of 1470 mW/cm^2^ ± 10%, tip diameter of 10 mm and a single, high-intensity continuous curing mode (3 M ELIPAR DEEPCURE, L1007-240 V INT, St. Paul, MN, USA).

### Pretreatment of prepared cavity before cementation

A selective etch protocol was employed using the universal adhesive following the manufacturer’s recommendations. The enamel margins of the cavities were selectively etched with 37% phosphoric acid gel (FineEtch 37%, Spident, Gojan-dong, Namdong-gu, Incheon, South Korea) for 20 s, followed by rinsing by air/water spray for 20 s, and finally drying with air for 5 s. Followed by application of generous coat of the universal adhesive prime and bond, the adhesive was actively rubbed on both enamel and dentin by a micro-brush (Easyinsmile, Passaic, New Jersey, USA) for 20 s then air thinning for 10 s and polymerized by for 20 s using the same curing unit.

### Luting procedure (Fig. 5)

**Fig. 5:**
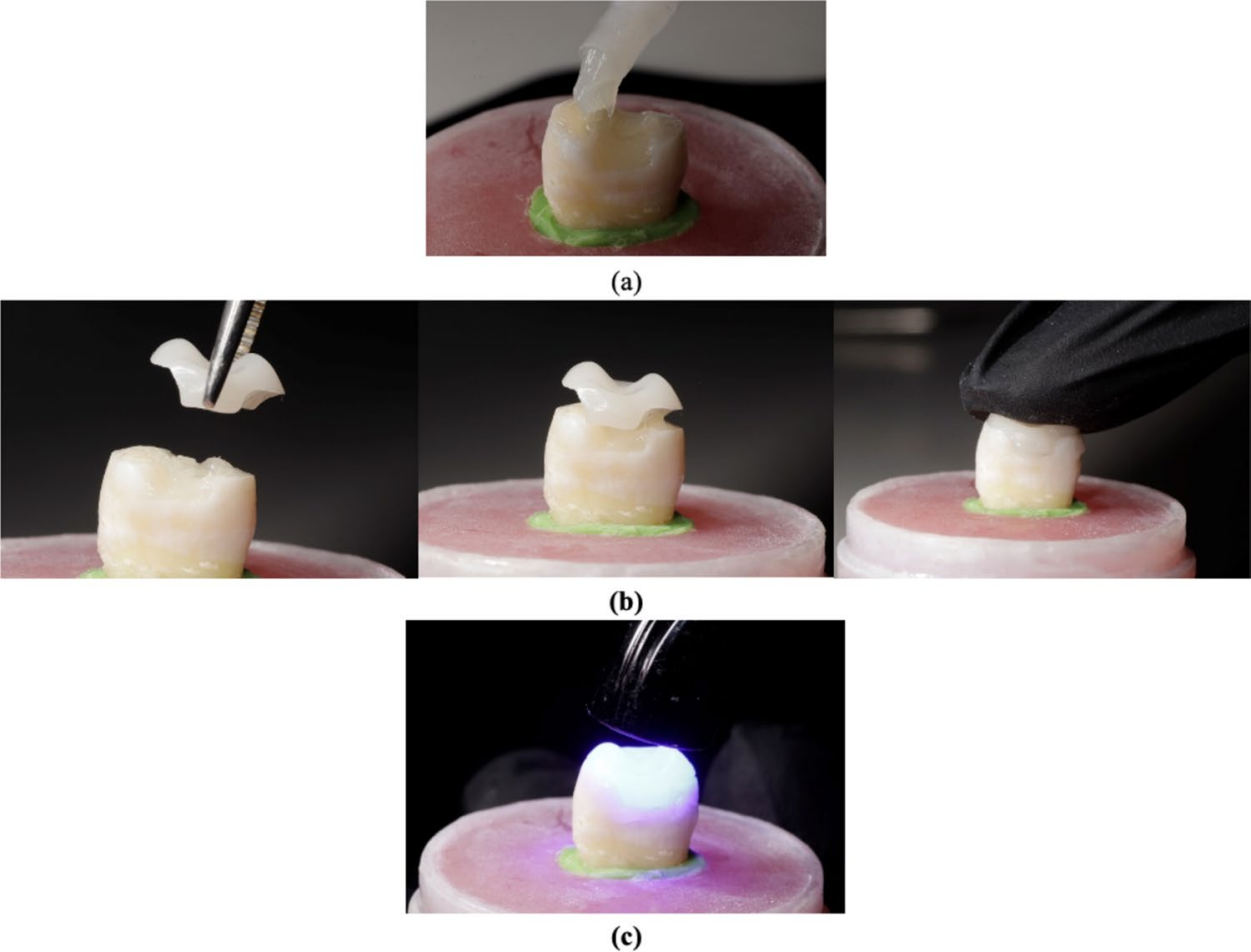
(a) Thin layer of the self-adhesive dual-cure resin cement, (b) CAD/CAM composite restoration placed with gentle finger pressure, (c) Polymerization using LED light curing unit

A thin layer of self-adhesive dual-cure luting resin (Calibra Universal, Dentsply Sirona) was applied to the surface of each pretreated cavity. Each CAD/CAM composite restoration was then positioned onto its corresponding cavity using gentle finger pressure, applied consistently by the same operator for all specimens. Excess resin was immediately removed using a micro-brush, and the tooth-restoration interface were coated with glycerin gel to serve as an air-blocking agent and prevent oxygen inhibition of the luting resin, ensuring complete curing. The luting resin was cured using the similar curing unit for 20 s from all directions. Once polymerization was complete, the gel was washed by air–water spray.

### Storage of specimens and thermocycling

Storage of all specimens in distilled water at room temperature for 24 h was done before thermocycling and fracture resistance testing. The cemented restorations were thermally cycled in distilled water for 5000 cycles at 5 ± 2 °C/55 ± 2 °C, with a 50 s dwell time and 10 s transfer time using a thermocycling device (SD Mechatronik Thermocycling, Julaba, Germany).

### Fracture resistance testing and failure mode evaluation

All specimens underwent fracture resistance testing using a Universal Testing Machine (Instron, Model 3345, Buckinghamshire, UK) [22]. Each specimen's acrylic block was fixed to the lower head of machine, while a continuous static load was applied to the crown using a five mm diameter stainless-steel ball attached to the upper movable head. The load was applied in an axial direction at a crosshead speed of 1.0 mm/min until failure occurred. The force required to induce failure (measured in Newton) was recorded using the machine’s software (Blue Hill Universal, Instron). Following fracture testing, the broken specimens were examined under a stereomicroscope (Nikon MA 100, Minato, Japan) to determine the mode of fracture. The fractures were categorized according to the following failure patterns: Type I: isolated fracture of the restoration; Type II: fracture involving a small portion of the tooth; Type III: fracture involving half of the tooth above the cementoenamel junction (CEJ); Type IV: fracture extending below the CEJ.

### Statistical analysis

Categorical data were expressed as frequencies and percentages and analyzed using the chi-square test. Numerical data were reported as means with 95% confidence intervals, standard deviations, and minimum and maximum values. Normality and homogeneity of variances were assessed using the Shapiro–Wilk and Levene's tests, respectively. While the data followed a normal distribution, the assumption of equal variances was not met. Therefore, Welch’s one-way ANOVA was applied, followed by the Games–Howell post hoc test. A significance threshold of *p* < 0.05 was used for all statistical tests. Analyses were conducted using R version 4.3.1 for Windows (R Core Team, 2023. *R: A language and environment for statistical computing*. R Foundation for Statistical Computing, Vienna, Austria. https://www.R-project.org/).

## Results

### Fracture resistance

Descriptive statistics and intergroup comparisons for of fracture resistance (N) for different groups is presented in Table 2. The fracture resistance showed significant differences between the experimental groups (*p* < 0.001). The highest fracture resistance was recorded in overlays with 2.5 mm of anatomical cusps reduction, followed by overlays with 2.5 mm of flat cusps reduction. This was followed by overlays with 1.5mm of anatomical cusps reduction, inlays, and overlays with 1.5mm of flat cusps reduction, respectively, with no significant differences between them (*p* > 0.05).

**Table 2:** Descriptive statistics and intergroup comparisons for of fracture resistance (N) for different groups

Group	Mean	95% confidence interval		SD	Min	Max
		Lower	Upper			
Inlay	1079.89^B^	977.72	1182.07	187.96	802.06	1351.01
Anatomical 1.5	1123.91^B^	999.29	1248.53	168.22	916.94	1392.38
Anatomical 2.5	1540.34^A^	1173.64	1907.05	495.02	894.58	2111.45
Butt joint 1.5	927.49^B^	855.75	999.23	96.84	769.29	1026.13
Butt joint 2.5	1293.77^AB^	1140.52	1447.02	206.88	994.90	1652.00

Different superscript letters indicate a statistically difference within the same horizontal row*; significant (*p* ≤ 0.05)

### Mode of failure

Intergroup comparisons, frequencies, and percentages of mode of failure for different groups are presented in Table 3. There were no significant differences between the groups regarding different modes of failure (*p* = 0.489).

**Table 3:** Intergroup comparison, frequencies and percentages values for mode of failure

Failure mode	*n*(%)					*p* value
	Inlay	Anatomical 1.5	Anatomical 2.5	Butt joint 1.5	Butt joint 2.5	
Isolated fracture of restoration (I)	3 (21.4%)	2 (28.6%)	5 (71.4%)	2 (28.6%)	2 (28.6%)	0.489ns
Fracture a small tooth portion (II)	3 (21.4%)	0 (0.0%)	0 (0.0%)	0 (0.0%)	1 (14.3%)	
Fracture half of tooth above CEJ (III)	5 (35.7%)	2 (28.6%)	1 (14.3%)	2 (28.6%)	3 (42.9%)	
Fracture below CEJ (IV)	3 (21.4%)	3 (42.9%)	1 (14.3%)	3 (42.9%)	1 (14.3%)	

ns; non-significant (*p* > 0.05)

## Discussion

The tooth’s integrity, morphology, and functionality must be restored and rebuilt by a high-quality restorative approach [23]. Partial coverage restorations, such as inlays and overlays, are conservative treatment strategies that cover the entire occlusal surface of the tooth or part of it. They can be implemented for conservative restoration of mutilated vital teeth or in cases of bruxism or involuntary clenching associated with occlusal tooth wear. For these types of restoration, bonded restorations, either ceramics or resin composite, as well as the suitable adhesive and luting resins, are recommended [24].

The objective of this study was to identify the most effective cavity preparation design for treating MOD cavities in vital maxillary premolars. An optimal restorative approach should strike a balance between preserving the function and aesthetics of the restored tooth while reducing the likelihood of future fractures. Inadequate cavity designs have been associated with poor stress distribution and an increased risk of fractures in both the tooth and the restoration [25].

According to the results of the present study, the null hypothesis was rejected, as the fracture resistance was influenced by the restoration design, form, and thickness. The overlay design associated with 2.5 mm of anatomic cusps reduction had the highest fracture resistance in comparison to all other groups.

This finding in accordance with several previous studies [1, 15, 25–28]. Overlays are reported to distribute the functional load over the entire occlusal surface rather than being concentrated in load bearing spots [29]. Therefore, they are often indicated to stabilize compromised teeth such as those that have undergone root canal treatment or have cracks [29]. On the contrary, the present findings disagree those of two other investigations [30, 31] in as far as the fracture resistance of inlays was higher than that of overlays. This difference could be attributed to the different types of materials (composite & feldspathic ceramic) used [30, 31].

Regarding the thickness of the restoration, the highest fracture resistance values were recorded when the amount of occlusal reduction was 2.5 mm. This finding agrees with previous results [8, 32]. It is expected that as the restoration thickness increase, the critical load required to break it will also increase. Another attribution is the anatomic form of the prepared occlusal surface. Anatomic cuspal reduction generally decreases the height of cusps with subsequent diminution of cuspal leverage with the applied stresses reducing its liability to fracture. [33, 34] In addition, the engagement of both cusps led to a balanced distribution of stresses within the cavity, so that the fracture resistance was higher in overlays than in inlays. Moreover, the use of anatomical occlusal preparation increased fracture resistance compared to flat preparation due to the subsequent improved stress and force distribution that follows the natural anatomy of premolars improving stability and resolution of compressive stresses. In addition, anatomic preparation provides sufficient thickness over the whole occlusal surface, avoiding stress concentration points at the different thicknesses of the restoration [35, 36]. On the other hand, the inlay preparation might induce wedging action between the cusps especially after thermocycling with its subsequent effect on the bonded restoration leading to early fracture of the tooth.

Early restoration failure is caused by an uneven transfer of force due to a variation in modulus between restorative materials and natural dentin. The value of the modulus of elasticity of dentin lies in the range of 10–20 GPa. The E-modulus of the Brilliant Crios composite is 10 GPa. The low E-modulus of this composite ensured shock absorption of the masticatory pressure and peak loads that occur can be well absorbed by the composite [37]. This allowed the material to deform to the same extent as the underlying dentin and, as a result, convey the applied load to the underlying dentin instead of building it up in the restoration [38]. Based on the statistical findings and observed fracture patterns in this in vitro study, all tested material thicknesses endured loads exceeding those commonly encountered in clinical scenarios. In addition, anatomic reduction designs with a minimum thickness of 2.5 mm are considered a safe option, as they help minimize the risk of non-restorable cusp fractures in cusp coverage restorations involving extensive MOD cavities. The results do not contraindicate the use of 1.5-mm thick overlays, but thicker overlays may be used for patients with high load requirements or whose teeth have been severely worn down or destroyed.

The limits of this study offer respected perceptions for a comprehensive clarification. First, the experiment includes inserting a rigid sphere into extracted teeth with MOD cavities. While this method is a simple evaluation of MOD cavities strength, it fails to fully account for the complicated interactions between horizontal and rotating stresses on cusp surfaces, as well as the complex dynamics of teeth occlusion and mandibular movements. Several additional factors, such as loading mode and direction, crosshead speed, and indenter size, may also impact fracture resistance. In addition, while controlled conditions were maintained, using recently extracted intact teeth distinctions with the dynamic nature of teeth mutilation often encountered in clinical cases [39].

## Conclusions

Within the limitations of the current study, we concluded that:

1. The CAD/CAM composite blocks are considered as promising materials in restoring premolars MOD cavities with or without cusp reduction.
2. The fracture resistance of the composite CAD/CAM fabricated restorations used in restoring maxillary premolars with MOD cavities is affected by the restoration design, thickness and cusps reduction form.
3. The overlay design in conjunction with anatomical cusps reduction of 2.5mm can reinforce maxillary premolars teeth with MOD cavities.

## Data Availability

Data are available upon reasonable request from the corresponding author.
